# Dual barrier system against xenomitochondrial contamination in mouse embryos

**DOI:** 10.1038/s41598-023-50444-2

**Published:** 2023-12-27

**Authors:** Masaya Komatsu, Hikaru Takuma, Shun Imai, Maiko Yamane, Masashi Takahashi, Takuto Ikegawa, Hanako Bai, Hidehiko Ogawa, Manabu Kawahara

**Affiliations:** 1 Laboratory of Animal Genetics and Reproduction, Research Faculty of Agriculture, Hokkaido University, Sapporo, Hokkaido, 060-8589 Japan; 2 Graduate School of Global Food Resources/Global Center for Food, Land and Water Resources, Hokkaido University, Sapporo, Hokkaido, 060-8589 Japan; 3 Department of Bioscience, Tokyo University of Agriculture, Tokyo, 156-8502 Japan; 4 Present Address: Hokkaido Agricultural Research Center, NARO, Sapporo, Hokkaido, 062-8555 Japan

**Keywords:** Developmental biology, Embryology

## Abstract

Heteroplasmic mammalian embryos between genetically distant species fail to develop to term, preventing transmission of xenomitochondrial DNA to progeny. However, there is no direct evidence indicating the mechanisms by which species specificity of the mitochondrial genome is ensured during mammalian development. Here, we have uncovered a two-step strategy underlying the prevention of xenomitochondrial DNA transmission in mouse embryos harboring bovine mitochondria (mtB-M embryos). First, mtB-M embryos showed metabolic disorder by transient increase of reactive oxygen species at the 4-cell stage, resulting in repressed development. Second, trophoblasts of mtB-M embryos led to implantation failure. Therefore, we tested cell aggregation with tetraploid embryos to compensate for the placentation of mtB-M embryos. The 14 mtB-M embryos harboring bovine mtDNAs developed to term at embryonic day 19.5. Taken together, our results show that contamination of bovine mtDNA is prohibited by embryonic lethality due to metabolic disruption and failure of placentation, suggesting these represent xenomitochondrial elimination mechanisms in mammalian embryos.

## Introduction

The transmission of genomic information from parents to progeny maintains the uniqueness of species. The two types of cellular DNA are stored in the nucleus and the mitochondria. While mitochondrial DNA (mtDNA) is uniform in size, structure, and composition in all mammals, species-specific mtDNA properties develop and adapt during evolution^1,2^. These properties may be acquired the habitat where the holder of the mitochondria lives sometime during its evolutionary history, which subsequently defines the species’ identity and nuclear DNA. The effects of xenogeneic mitochondria on mammalian development have been widely examined^3–5^.

We have previously analyzed the developmental competence of mouse embryos harboring bovine mitochondria (mtB-M embryos)^6^. The model system for mtB-M embryos would be a measure to elucidate the effects of bovine mtDNAs derived from extremely evolutionarily distant species on the development of mice with mtDNA heteroplasmy. Cattle and mice have evolved in entirely different ways and their differences are significant. In our previous study, inter-order mtB-M embryos showed a much lower developmental rate up to the blastocyst stage than in vitro fertilized (IVF) embryos^6^. Additionally, mtB-M embryos did not implant successfully after embryo transfer^6^. These results suggest that the heteroplasmic state disturbs both pre- and post-implantation development. Indeed, it may be necessary to inhibit the development of xenomitochondrial embryos to preserve species-specific mtDNA in the context of heredity. However, the mechanism by which the pre- and post-implantation development of mtB-M embryos is blocked remains unknown. Identifying the barrier(s) in the development of mtB-M embryos will demonstrate significance of the intraspecific genetic specificity of embryonic mtDNA in mammalian ontogeny.

## Results

### Early development of mtB-M embryos

First, we analyzed the potential cause of implantation failure in mtB-M embryos. In accordance with our previous report^6^, we prepared mtB-M embryos from mouse embryos via cell fusion with the mitochondria-enriched region of bovine embryos (Movie S1). While some of the mtB-M embryos developed to the blastocyst stage, none of the embryos developed to term after embryo transfer. In contrast, the control embryos, normal IVF embryos, mtM-M embryos (mouse embryos with mitochondria derived from another mouse embryo), and non-mtB-M embryos (mouse embryos containing bovine cytoplasm distinct from the mitochondria enrichment region from bovine embryos) all developed to term (Table S1). These results suggested that the rate of development to term decreased following the introduction of bovine mtDNA into mouse embryos, as exemplified by the mtB-M embryos.

**Figure 1:**
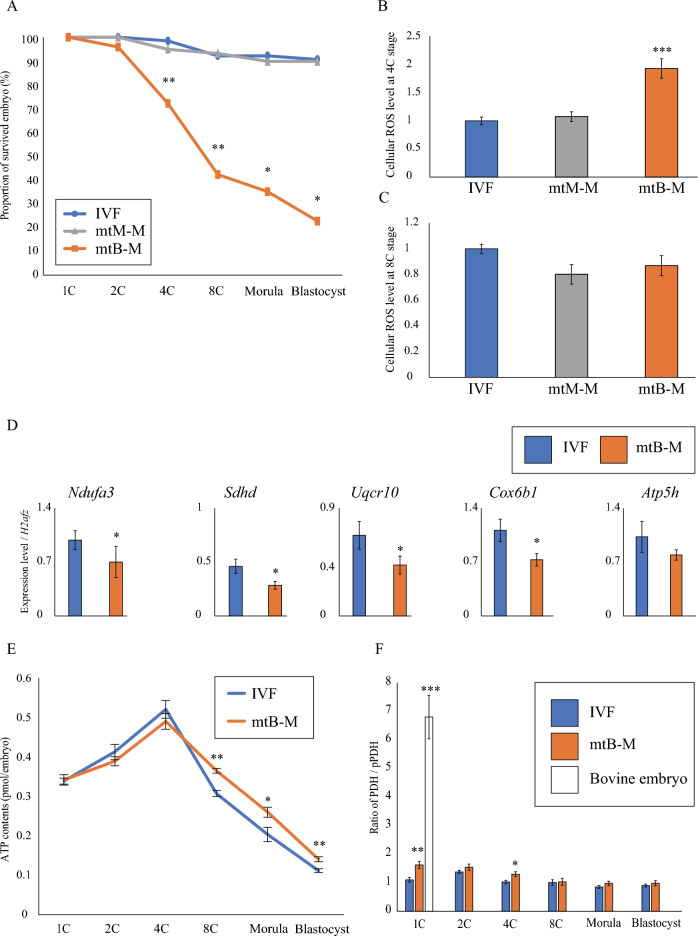
Preimplantation development and metabolic profile of mouse embryos harbouring bovine mtDNAs. (**A**) Developmental arrest in the mouse embryos harbouring bovine mtDNAs (mtB-M embryos (n = 97); orange). Note that proportions of surviving embryos in mtB-M embryos from the 4-cell (4C) stage were significantly reduced compared to IVF embryos (n = 128; blue) and mtM-M embryos (mouse embryos with mitochondria derived from other mouse embryos (n = 59); grey). Data were analyzed after arcsine transformation. *p < 0.05 and **p < 0.01. (**B, C**) Production levels of cellular reactive oxygen species (ROS) at the 4C stage and 8C stage. IVF (4C: n = 23, 8C: n = 23), mtM-M (n = 4, 12), and mtB-M (n = 7, 18). The ROS level in the mtB-M embryos at the 4C stage was elevated compared to that in the IVF embryos (***p < 0.001). Bar graph shows mean ± standard error of the mean (S.E.M.). (**D**) Relative expression of mitochondrial respiratory chain (MRC) complex-related genes at the 4C stage. *Ndufa3* for MRC complex I [IVF (n = 6) vs. mtB-M (n = 7)], *Sdhd* for MRC complex II (n = 4 vs. n = 4), *Uqcr10* for MRC complex III (n = 6 vs. n = 7), *Cox6b1* for MRC complex IV (n = 6 vs. n = 7), and *Atp5h* for MRC complex V (n = 4 vs. n = 4). Each value was normalised to *H2afz* expression. *p < 0.05. (**E**) The ATP contents (pmol / embryo) in IVF and mtB-M embryos. More than 50 embryos were measured for each cell stage, and then, values within mean ± 1 standard deviation were selected. Among them, 28 to 42 embryos were analyzed for each cell stage. The data for all embryos were represented in Fig. S2. *p < 0.05 and **p < 0.01. (**F**) The PDH activity in IVF (n = 18–33; blue), mtB-M (n = 15–21; orange) embryos, and presumptive 1C stage bovine (n = 9; white) embryos. *p < 0.05, **p < 0.01, and ***p < 0.001.

We further determined the developmental stage at which the in vitro development to mtB-M embryos was terminated (Fig. 1A). As a result, the proportion of surviving embryos to all cultured embryos significantly decreased from the 4-cell stage (*p* < 0.05). Additionally, the production of reactive oxygen species (ROS) in the mtB-M embryos at the 4-cell stage was also significantly higher than that in the IVF and mtM-M embryos at that stage (*p* < 0.001) (Fig. 1B and Fig. S1A), whereas by the 8-cell stage, there were no significant differences (Fig. 1C and Fig. S1B). Moreover, most of the mRNA expression levels of representative genes related to the mitochondrial respiratory chain (MRC) complexes were significantly downregulated in mtB-M embryos compared to those of the others (*p* < 0.05) (Fig. 1D). Additionally, the ATP content is significantly higher in mtB-M embryos than in IVF embryos at the 8-cell (*p* < 0.01), morula (*p* < 0.05), and blastocyst (*p* < 0.01) stages (Fig. 1E and Fig. S2), suggesting that the increased ROS production that occurred at the 4-cell stage caused metabolic dysfunction, including excessive ATP production. To further explore the potential cause of metabolic dysfunction in the mtB-M embryos, we investigated pyruvate dehydrogenase (PDH) activity by evaluating the ratio of PDH to phosphorylated PDH, its inactive form (Fig. 1F and Fig. S3). Resultantly, PDH activity in mtB-M embryos was significantly higher than that in IVF embryos at the 1-cell (*p* < 0.01) and 4-cell (*p* < 0.05) stages (Fig. 1F). This is likely due to the introduction of mitochondria derived from bovine embryos because the PDH activity in 1-cell bovine embryos was markedly higher than that in both IVF mouse embryos (*p* < 0.001). Thus, these results show that the introduction of bovine mitochondria with markedly-high PDH activity into mouse embryos induces metabolic dysfunction and subsequently embryonic arrest, suggesting that it is the first barrier for xenomitochondrial DNA transmission to progeny in mice.

### Trophectoderm cells of mtB-M embryos

**Figure 2:**
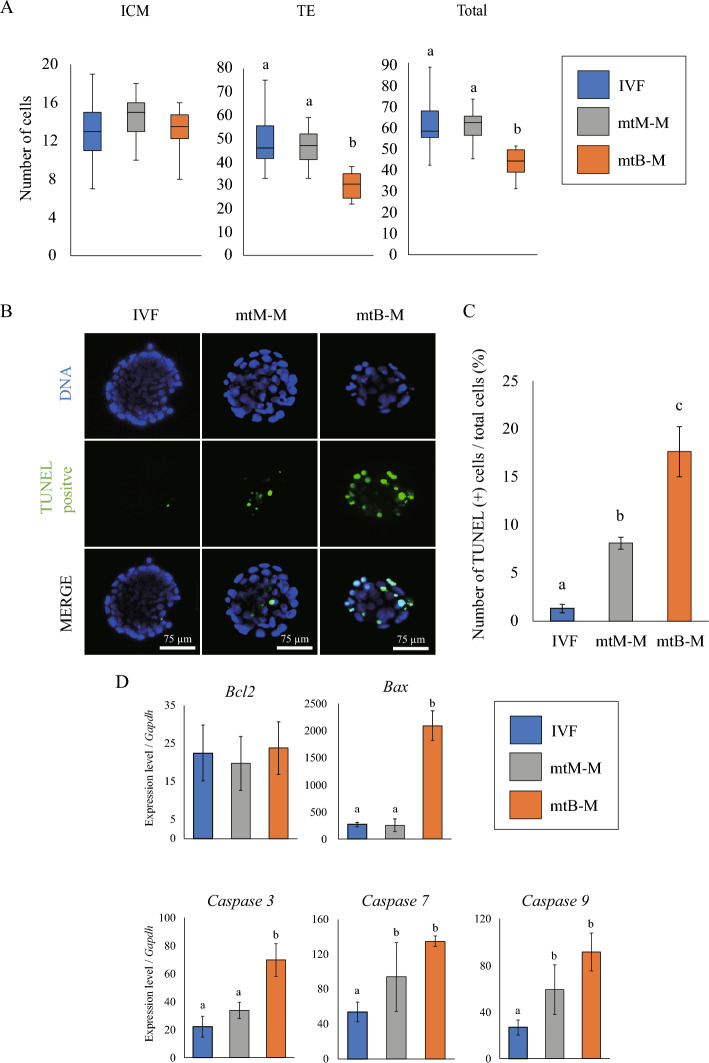
Trophectoderm of mouse blastocysts harbouring bovine mtDNAs. (**A**) ICM/TE cell allocation in mtB-M blastocysts. Boxplots represent ICM and TE cell numbers based on OCT3/4 and CDX2 immunostaining and total cell number. IVF control (blue), mtM-M (mouse embryos with mitochondria derived from other mouse embryos; grey), and mtB-M (mouse embryos with mitochondria derived from a bovine embryo; orange). IVF, n = 47; mtM-M, n = 9; mtB-M, n = 6. ^a,b^Different letters denote significant differences (*p* < 0.05). (**B**) TUNEL assay for detecting DNA fragmentation in IVF control, mtM-M, and mtB-M embryos. (**C**) Bar graph showing mean ± S.E.M. of the ratio of TUNEL-positive cells to the total cells. IVF, n = 6; mtM-M, n = 19; mtB-M, n = 5. ^a-c^Different letters denote significant differences (*p* < 0.05). (**D**) Relative expression levels of five representative apoptosis-related genes (*Bcl2, Bax, Caspase 3, 7,* and *9*) in mouse IVF embryos (blue), mtM-M embryos (grey), and mtB-M blastocysts (orange). Each value was normalised to *Gapdh* expression. Ten blastocysts were collected per sample, and three independent samples were analysed. ^a,b^Different letters denote significant differences (*p* < 0.05).

Next, we analyzed the cell specialization of the mtB-M blastocysts. Immunostaining for the representative marker proteins, OCT3/4 for the inner cell mass (ICM) and CDX2 for the trophectoderm (TE), revealed a TE cell-dominant decrease in mtB-M blastocyst cell populations (Fig. 2A). In agreement with TE cell-dominant cell death in mtB-M blastocysts, fluorescent signals derived from the transferase-mediated dUTP nick end-labeling (TUNEL) assay were detected, particularly in the outermost cells (Fig. 2B and C). We confirmed that the mRNA expression of representative pro-apoptotic genes increased in mtB-M blastocysts (Fig. 2D).

**Figure 3:**
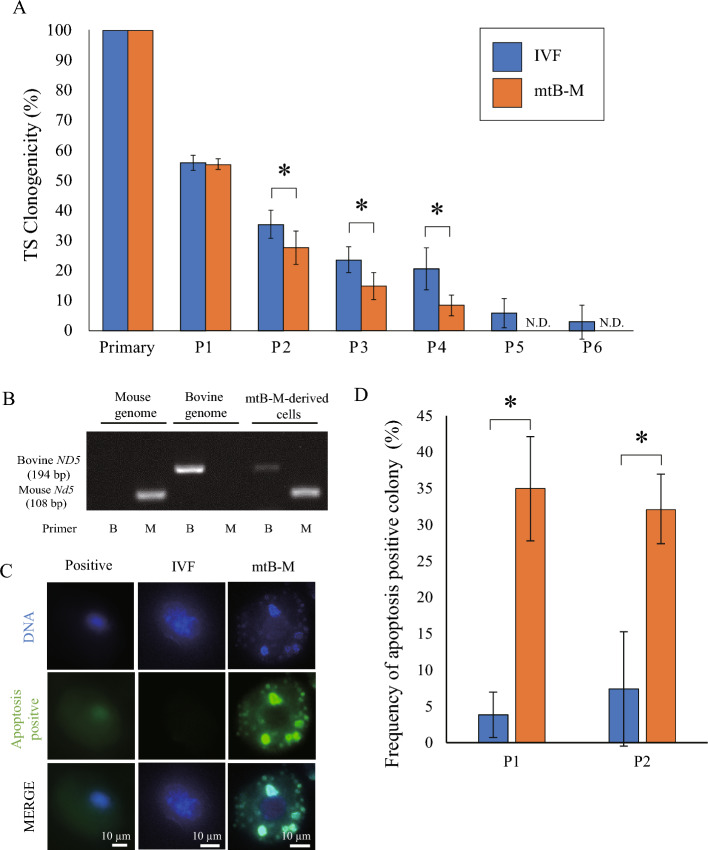
Effects of contamination of bovine mitochondria on clonogenicity TS cells derived from TE. (**A**) The clonogenicity is shown as the ratio of the number of formed colonies to the total number of embryos subjected to the primary culture at every passage. Bar graph represents mean ± S.E.M.. IVF embryo (n = 34). mtB-M embryo (n = 55). *p < 0.05. (**B**) Genotype analysis by PCR with species-specific *ND5* primers in TS like cell colony (P3). Primer M and B mean mouse *Nd5* and bovine *ND5*, respectively. (**C**) CellEvent *Caspase3/7* stain for detecting apoptosis positive cell in TS like cell colony (P1). (**D**) The bar graph shows the frequency of apoptosis positive colony in P1 & 2. Bar graph represents mean ± S.E.M.. P1: IVF embryo (n = 26). mtB-M embryo (n = 20), P2: IVF embryo (n = 27). mtB-M embryo (n = 53). *p < 0.05.

To assess the ability of TE differentiation mtB-M blastocysts, we established trophoblast stem (TS) cells from mtB-M blastocysts. Although mtB-M-derived cells showed outgrowth on feeder cells, no colonies derived from mtB-M blastocysts survived after passage 5 (P5), unlike the control colonies from wild-type (WT) IVF blastocysts (Fig. 3A). Bovine mitochondria were detected during the establishment of TS cells (Fig. 3B). Since colony maintenance after P5 is generally required for TS cell stabilization^7^, this result shows that mtB-M-derived cells cannot form TS cells. Furthermore, in TS-like cells derived from mtB-M embryos (P1 and P2), the number of colonies with apoptotic cells significantly increased (Fig. 3C and D). These results indicate that peri-implantation lethality in mtB-M embryos is mainly caused by trophectodermal defects and apoptosis.

### Compensation of placentation in mtB-M embryos by tetraploid embryos

**Figure 4:**
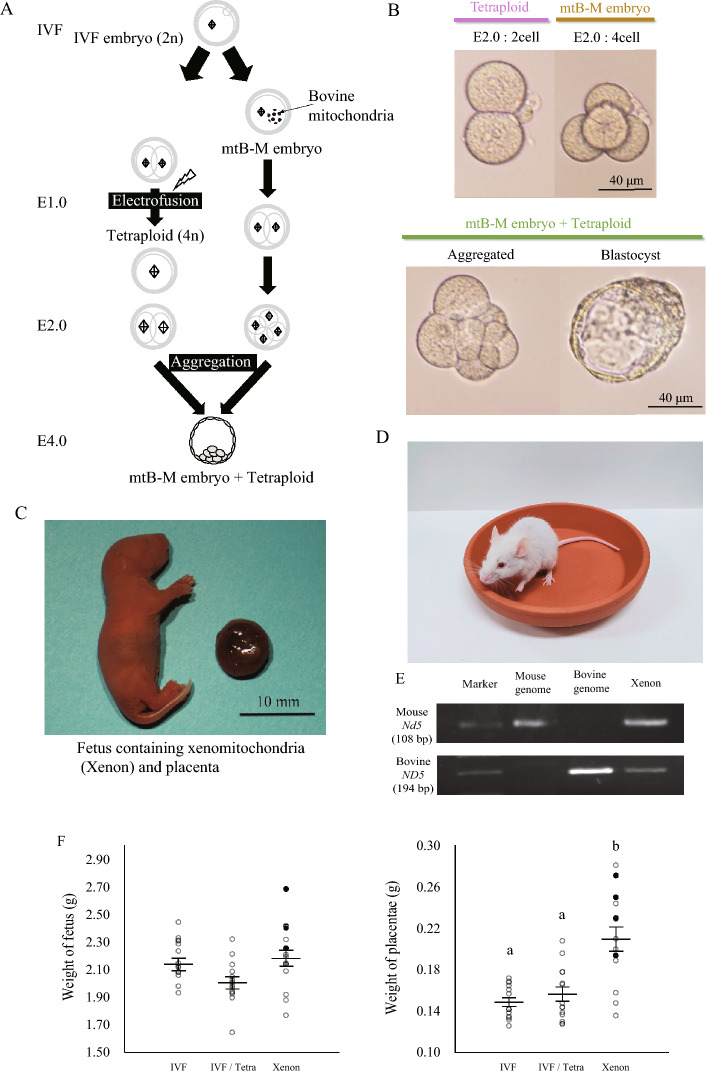
Generation of xenomitochondrial mice by aggregation with tetraploid embryos. (**A**) Schematic illustration for the production of mtB-M embryos aggregated with tetraploid embryos. (**B**) The images of embryo aggregation of mtB-M embryos with tetraploid embryos. (**C**) Representative pup and placenta derived from an mtB-M embryo aggregated with a tetraploid embryo. We named these pups “Xenon.” (**D**) 4-week-old Xenon mouse. (**E**) Genotype analysis by PCR with species-specific *ND5* primers showing that the Xenon pups contain bovine mtDNAs. The markers in the upper low and lower low indicate 100 bp and 200 bp, respectively. (**F**) The body and placenta weights of Xenon pups (n = 14) at birth. White circle: alive at birth with spontaneous respiration; black circle: dead at birth. Data represent the mean ± S.E.M.. IVF embryo-derived (n = 14). IVF/tetraploid embryo-derived (n = 14). ^a,b^Different letters denote significant differences (*p* < 0.05).

Trophectoderm defects may be a second barrier for xenomitochondrial DNA transmission to progeny by inhibiting embryonic development during the peri-implantation period. To restore the trophoblast disorder observed in mtB-M embryos, we aggregated mtB-M embryos with IVF-derived tetraploid embryos, as previously described^8^ (Fig. 4A and B, and Movie S2). No tetraploid embryos developed beyond embryonic days 10.5 (E10.5)^9^. In total, 158 blastocysts derived from mtB-M embryos aggregated with tetraploid (mtB-M/tetraploid) embryos were transferred to the uteri of 10 pseudo-pregnant females. Among these 14 live pups were collected at E19.5 (Table S2). The pups exhibited normal neonatal morphology and growth (Fig. 4C and D). The pups, xenomitochondrial mice harboring bovine mitochondria, were termed “Xenon” mice, which is derived from the Greek term “Xenos” defined as “a foreigner.” Polymerase chain reaction (PCR) analysis was performed using total DNA collected from neonatal fingertips and primers specific for bovine mitochondrial *ND5*. The recovered Xenon pups possessed bovine *ND5*, demonstrating that they were derived from the mtB-M/tetraploid embryos (Fig. 4E). The PCR amplicons were sequenced and aligned to the bovine mitochondrial *ND5* sequence (RefSeq ID: NC_006853.1). As expected, unlike neonates, bovine *ND5* amplicons were not detected in the DNAs from any of the nine Xenon placentas at E19.5. The mean body weight of the 14 Xenon fetuses was 2.180 ± 0.063 g, which was similar to that of the IVF and IVF/tetraploid controls (2.175 ± 0.039 g and 2.010 ± 0.042 g, respectively; Fig. 4F). Among the 14 Xenon fetuses, four (28.6%) did not show spontaneous respiration (black circles in Fig. 4F). The mean placental weight of the 14 Xenon fetuses was 0.210 ± 0.012 g, which was significantly more than those of the IVF and IVF/tetraploid controls (0.149 ± 0.004 g and 0.157 ± 0.007 g, respectively; Fig. 4F). Among the Xenon pups showing spontaneous respiration, four were nursed by foster mothers. These pups survived for more than one year. These results demonstrate that compensation of placentation in mtB-M embryos by tetraploid embryos drastically improve the post-implantation development in the mtB-M embryos, suggesting that the trophoblast disorder resulting from xenomitochondrial contamination is the second barrier to prevent xenomitochondrial DNA transmission to the progeny.

### Transmission of bovine mtDNA to fetal-lineage cells in Xenon mice

**Figure 5:**
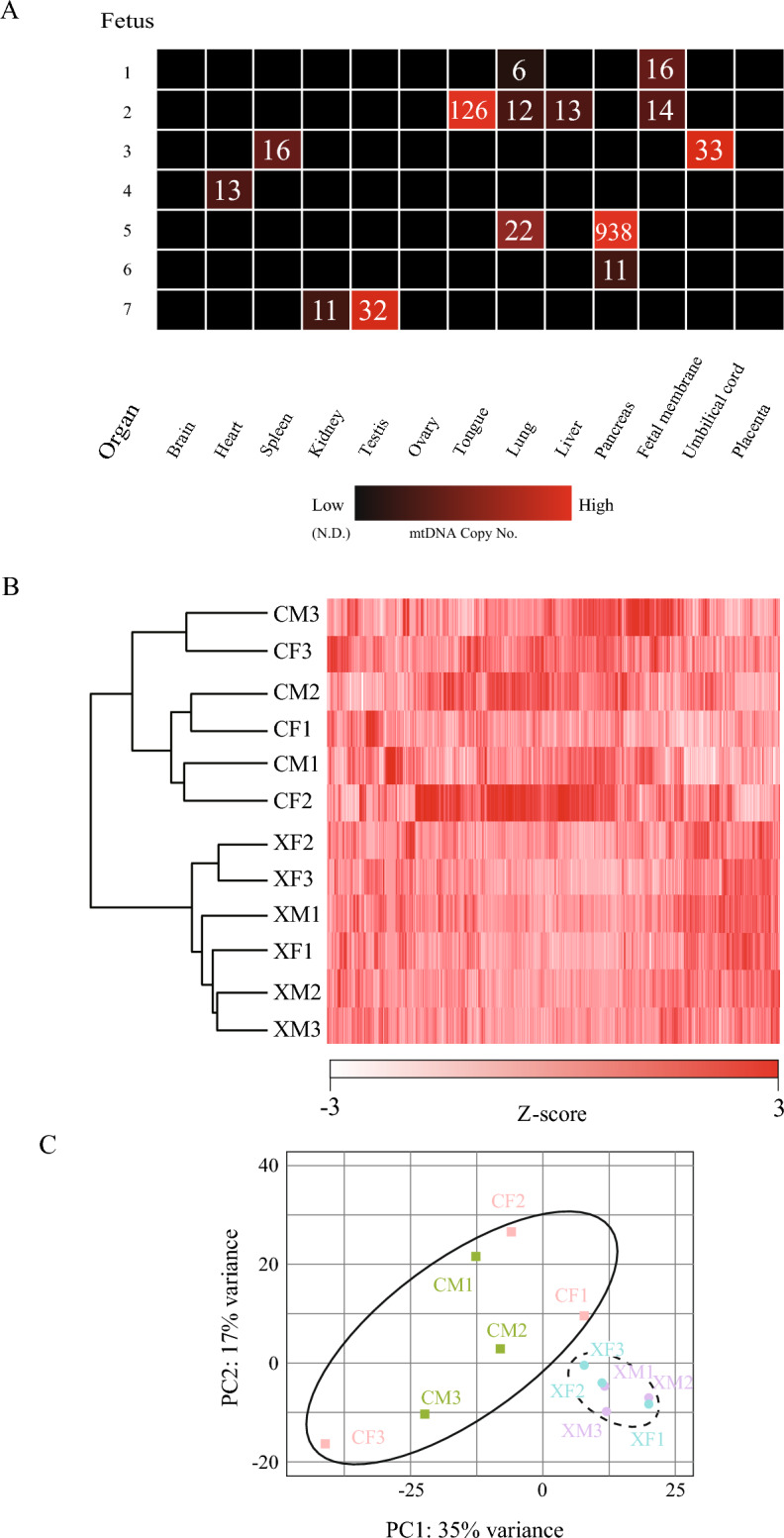
Transcriptomic profile of Xenon lungs harbouring bovine mtDNAs. (**A**) Heatmap of bovine mtDNA copy number in major tissues from Xenon pups. Heatmap showing the bovine mtDNA copy number in total DNA (100 ng) derived from each organ of Xenon pups at E19.5 (n = 7). The colour scale represents the mtDNA copy number from red (high; 1000 copies) to black (not detected). (**B**) Heatmap with hierarchical clustering of all samples from IVF controls and Xenon lungs at E19.5. All gene expression levels were converted to Z-scores, which were calculated by comparing the fragments per kilobase of exon per million mapped reads (FPKM) values. CM, IVF control male; CF, IVF control female; XM, Xenon male; XF, Xenon female. The color scale represents the Z-score from red (maximum: 3) to white (minimum: − 3). Among the 6 Xenon lungs used for RNA-seq, XM1 (ID#1 in **A**) was derived from an individual without spontaneous breathing. (**C**) Principal component analysis of gene expression in control lungs from IVF embryos and Xenon lungs at E19.5.

**Figure 6:**
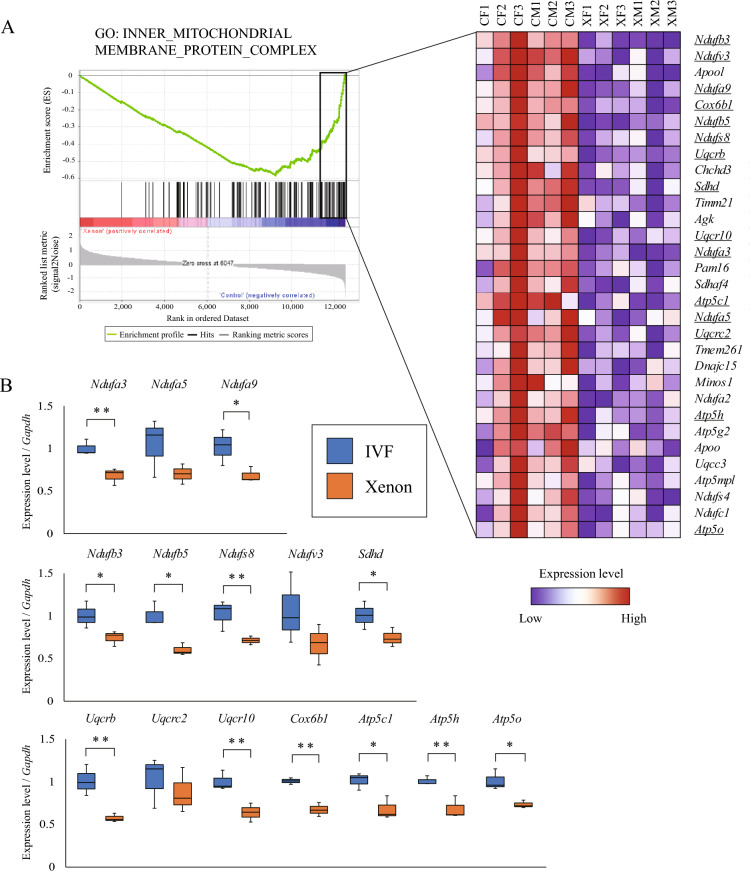
Downregulation genes related to mitochondrial respiratory chain complex in Xenon lungs. (**A**) Enrichment plot for the selected GO term, INNER_MITOCHONDRIAL_MEMBRANE PROTEIN_COMPLEX showing the running Enrichment Scores (ESs) in each gene ranked by expression level (bar-code lines). The bottom portion represents the value of the ranking metric moving down the list of ranked genes. The right heatmap shows the gene expression levels in the leading-edge subset of the left panel. (**B**) Relative expression levels of genes encoding MRC complexes I to V subunits were determined by qPCR. The genes examined correspond to the underlined gene names shown in the heatmap in (**A**). *p < 0.05, **p < 0.01.

To investigate the effects of bovine mtDNA on fetal-lineage cells, we measured the weights of the major organs collected from Xenon mice. The organ weights in Xenon mice were similar to those in WT control mice derived from IVF embryo transfer (Fig. S4A). To investigate the distribution of bovine mtDNA in Xenon mice, we performed quantitative real-time PCR (qPCR) using primers specific for the bovine *ND5* gene in their primary organs: the brain, heart, spleen, kidney, testis, ovary, tongue, lung, liver, pancreas, fetal membrane, and umbilical cord (Fig. 5A). The qPCR results showed that heterogeneous bovine mtDNA was randomly distributed throughout the whole body, except for within the brain and ovaries, in Xenon mice. We did not detect any bovine mtDNA in the placenta. Next, we focused on high-frequency postnatal death by respiration failure in Xenon mice at E19.5 (Fig. 4F). As the lungs are the primary organs of the respiratory system in mammals, we performed histological evaluations following hematoxylin–eosin staining of the left lungs of Xenon mice. There were no remarkable histological abnormalities in the alveolar lumen area, the number of alveoli, or the area of clara cells, which protect the respiratory tract^10^ per unit area, in Xenon mice compared to WT controls (Fig. S4B and C). Additionally, we compared the transcriptome of 6 Xenon lungs to those of the 6 WT lungs. In addition to the 4 Xenon lungs (fetus ID#1, 2, 5, and 6) in Fig. 5A, further 2 Xenon lungs were prepared for the transcriptome analysis after detection of bovine mtDNAs by nested PCR (Fig. S5). Although we could not detect bovine mtDNAs in the lung of ID#6 fetus in Fig. 5A by qPCR, bovine mtDNAs in the ID#6 lung was detected by the more sensitive nested PCR. Xenon mouse lungs were subjected to global gene expression analyses using RNA sequencing (RNA-seq) after the detection of bovine mtDNA (XM1-3 and XF1-3 in Fig. 5B). RNA-seq revealed a unique transcriptome in the Xenon mouse lung that differed from that of the controls. Heat mapping with hierarchical clustering (Fig. 5B) and principal component analysis (Fig. 5C) indicated that sex differences had an insignificant effect on the transcriptome within each category. Next, we attempted to align the RNA-seq reads to a known bovine mtDNA genome sequence (RefSeq ID: NC_006853.1). Interestingly, no matching reads were found in any of the examined Xenon mouse samples (Table S3), indicating that bovine mtDNA transcription was silenced in Xenon mouse lungs. Furthermore, all transcripts expressed in the Xenon mouse lung were characterized using Gene Ontology (GO) and gene set enrichment analyses (GSEA). Most transcripts contained in the inner mitochondrial membrane protein complex (GO ID:0098800) were downregulated in Xenon mouse lungs (Fig. 6A). The expression levels of 15 genes encoding mitochondrial respiratory chain complex proteins essential for oxidative phosphorylation were analyzed using qPCR. Of these, 12 genes were significantly downregulated in the Xenon mouse lung samples, including components of MRC complex I: *Ndufa3*, *Ndufa9*, *Ndufb3*, *Ndufb5*, and *Ndufs8*; MRC complex II: *Sdhd*; MRC complex III: *Uqcrb* and *Uqcr10*; MRC complex IV: *Cox6b1*; and MRC complex V: *Atp5c1*, *Atp5h*, and *Atp5o* (Fig. 6B). These results indicated that bovine mtDNA introduced into mouse embryos induces global transcriptomic alterations, including altered MRC complex-encoding genes in mouse nuclear DNA in the lungs.

## Discussion

Accurate transmission of species-specific genetic materials to progeny is a fundamental issue for continued existence of the species. Even though mtDNA carries much less information than nuclear DNA, it is important that mtDNA transmits species-specific genetic information because it distinguishes a species from other species and characterizes it. This study demonstrates a robust exclusion mechanism against xenomitochondrial DNA contamination in mammalian embryos.

We first observed that the mtB-M embryos with bovine mtDNA underwent developmental arrest after the 2-cell stage (27/90), and thereby, the developmental rate to the blastocyst stage decreased to less than 40% (Fig. 1A). The mtB-M embryos at the 4-cell stage showed significantly higher ROS level with downregulation of respiratory chain complexes-related genes (Fig. 1B and D), followed by elevated ATP contents after the 8-cell stage (Fig. 1E). The diseased metabolic state in the mtB-M embryos was induced by introduction of bovine mitochondria because the PDH activity of bovine 1-cell embryo was tremendously higher than that of intact IVF mouse 1-cell embryo (Fig. 1F). Furthermore, the PDH activities of the mtB-M embryos at the 1-cell and 4-cell stages were significantly higher than those of the IVF embryos. These results indicate that incompatibility in metabolic state of mitochondria between different species induces developmental arrest, contributing to prevention of preimplantation development in the mtB-M embryos.

We next analyzed the TE of mtB-M embryos to explore the cause of postimplantation lethality^6^. The cell number of the mtB-M TEs was significantly decreased compared to that of the ICM because of increased apoptosis. This was supported by an increased frequency of apoptotic TS-like cell colonies derived from the mtB-M blastocysts. The difference in the response to xenomitochondria introduction between the TE and ICM may be related to the higher energic metabolism in TEs, which produce approximately 80% of ATP consumed in the whole blastocysts^11^, and the mtDNA replication in the stage that initiates only in TE^12^.

It is believed that eukaryotic cells have incorporated mitochondria arisen from α-proteobacteria into their cells. During evolution, ancestral mtDNA gradually adapted and gained functional and evolutionary specificity with respect to the environment where the individual thrives. In the lungs of Xenon mice harbouring bovine mtDNA, transcription from bovine mtDNA was completely silenced, indicating that mouse cells are not suitable for the transcription of bovine mtDNA. However, mouse embryos allowed bovine mtDNA to amplify and propagate around fetal tissues at random. Particularly, Xenon mouse lungs showed global changes in transcription associated with mitochondrial functions, including respiratory chain complexes. This disturbed gene expression pattern might be caused by contamination of bovine mtDNA. Transcription from bovine mtDNAs was prohibited in mouse cells (Table S3) and remained dysfunctional. Dysfunctional mtDNAs impair their transcriptional activity at the cellular level and lead to various severe disorders in human^13,14^.

Since transmission of the species-specific genetic materials to progeny is the principle of heredity, xenomitochondrial DNA contamination probably provides detrimental effects on the inherited individuals such as respiratory failure with disrupted lung transcriptome. However, effects of contamination of xenogeneic mtDNA on pre- and post-implantation development have not been examined in detail. Particularly, phenotypes of specific organs of fetuses carrying xenomitochondrial DNAs have been unexplored. In this context, analyses using mtB-M embryos provide novel insights into the maintenance of the genetic identity of mtDNA conspecific to the nuclear DNA. Certainly, xenomitochondrial contamination was likely to impair the viability of progeny. The transmission of bovine mtDNA into Xenon lung tissues causes high-frequency respiratory failure during the postnatal period. This study demonstrates that the genetic individuality of mtDNA is solidly protected from risk of xenogeneic contamination by a two-step strategy: metabolic disruption in preimplantation development and failure of placentation in post-implantation development.

## Methods

### Ethical approval

All animal experiments were approved by the Regulatory Committee for the Animal Care and Use of Animals of Hokkaido University and were performed in accordance with the National University Corporation Hokkaido University Regulations on Animal Experimentation (Approval No. 19-0162).

### In vitro preparation of bovine and mouse embryos

Preimplantation mouse and bovine embryos were produced using IVF, as previously described^6^, with slight modifications. All experiments used mice of the Slc:ICR strain (Sankyo Labo Service Co., Inc., Tokyo, Japan) between 8 and 12 weeks of age. Superovulation was induced in female ICR mice by injecting 7.5 IU pregnant mare serum gonadotropin (ASKA Pharmaceutical Co., Ltd., Tokyo, Japan) and 7.5 IU human chorionic gonadotropin (ASKA Pharmaceutical Co., Ltd.), administered 48 h apart. Sperm was collected from the cauda epididymis of male ICR mice, suspended in a 200 μL drop of human tubal fluid medium^15^ in paraffin oil, and pre-incubated for 90 min in a 5% CO_2_ atmosphere at 37 °C. Oocytes at metaphase II were collected from the murine oviducts 16 h after equine chorionic gonadotropin administration and transferred to a 100 μL drop of human tubal fluid medium containing 0.5–1 × 10^6^ sperm/mL. At 4–6 h after insemination, the embryos were washed with M2 medium^16^ to remove cumulus cells and then transferred to a drop of M16 medium^17^. Embryos were cultured in M16 until the blastocyst stage and used for subsequent experiments. Embryonic day 0 (E0) was defined as the time at which in vitro culture was started.

Bovine embryos were prepared by in vitro oocyte maturation, IVF, and subsequent in vitro culture. Briefly, cumulus-oocyte complexes were collected from slaughterhouse-derived ovaries and matured by culturing in TCM-199 medium (Thermo Fisher Scientific, MA, USA) at 38.5 °C in a humidified atmosphere of 5% CO_2_ for 22–24 h. In vitro-matured oocytes were transferred to Brackett and Oliphant medium^17^ containing 2.5 mM theophylline (FUJIFILM Wako Pure Chemical Corporation, Osaka, Japan) and 7.5 μg/mL heparin sodium salt (Nacalai Tesque, Kyoto, Japan). Frozen-thawed semen was centrifuged at 600 × *g* for 7 min in Brackett and Oliphant medium, and spermatozoa were added to the cumulus-oocyte complexes at a final concentration of 5 × 10^6^ cells/mL. After incubation for 18 h, the presumptive IVF zygotes were denuded by pipetting and cultured in mSOFai medium^18,19^ at 38.5 °C in a humidified atmosphere of 5% CO_2_ and 5% O_2_ in air. The start of insemination was regarded as day 0 (D0) of in vitro culture.

### Construction of mitochondrial hybrid mouse embryos harboring bovine mitochondria (mtB-M embryos)

All procedures for mtB-M embryo construction were performed as previously described^6^. Mouse embryos harboring bovine mitochondria were generated using an inverted microscope (Nikon Corporation, Tokyo, Japan) equipped with a set of micromanipulators and microinjectors (Narishige, Tokyo, Japan). Briefly, bovine embryos were centrifuged at 10,000 × *g* at 35 °C for 15 min in mSOFai medium containing 7.5 μg/mL cytochalasin B. The zona pellucida near the mitochondria-enriched region was cut with a glass knife. The bovine mitochondrial-enriched region was suctioned into a glass pipette with an equal volume of inactivated hemagglutinating virus of Japan (Ishihara Sangyo Kaisha, Ltd., Osaka, Japan) solution and wrapped in a embryo whose zona pellucida was also cut, and the polar bodies were removed (Movie S1). All mouse embryos were cultured in M2 medium supplemented with 7.5 μg/mL cytochalasin B and 0.1 μg/mL colcemid (FUJIFILM Wako Pure Chemical Corporation). For experimental and developmental controls, we prepared mtM-M. These mouse embryos were washed in M16 medium droplets and incubated at 37 °C for 60 min in a humidified atmosphere containing 5% CO_2_. Embryos were cultured to the four-cell stage for aggregation with tetraploid embryos.

### Assessments of metabolic states

Reactive oxygen species (ROS), cellular ATP content, and PDH activity were evaluated to characterize the mitochondrial metabolic states in mtB-M embryos compared with those in IVF embryos. These assays were conducted as previously described^20–23^. ROS in embryos were measured with a ROS sensitive fluorescent probe (CellROX® Green Reagent; Invitrogen, Carlsbad, CA, USA). ATP content of embryos was measured with a CellTiter-Glo® 2.0 Cell Viability Assay (Promega, WI, USA) using the luciferin-luciferase reaction. Each embryo was transferred at a time to sample tubes containing 50 µL MilliQ water, heated at 95 ˚C for 20 min, and stored at  − 80 °C until measurement. The samples were thawed on ice and placed in 96-well plates (Perkin Elmer, Waltham, MA, USA). Then, 50 μL of CellTiter-Glo® 2.0 was dispensed into each well. After reacting under light-shielded conditions at room temperature for 10 min, the luminescence intensity was measured using a luminometer (ARVO×4) (Perkin Elmer). Calibration curves were generated using ATP solutions ranging from 0 to 40 nM, and more than 50 embryos were measured for each cell stage (Fig. S2). Values within mean ± 1 standard deviation were selected and analyzed for each cell stage. PDH activity was measured via image analysis of immunostained slides. Primary antibodies [mouse anti-PDH E1 alpha, ab110334 (Abcam, Cambridge, UK) for PDH and rabbit anti-phospho-PDH E1alpha (S293), AP1062 (Merck Millipore, MA, USA) phosphorylated PDH (pPDH)] were used at 4 °C for 16 h. The reactions for the secondary antibody were carried out using a goat anti-mouse IgG (H + L) Cross-Adsorbed Secondary Antibody, Alexa Fluor 488, and A11001 (Invitrogen) for 30 min at room temperature under light-shielded conditions. Counter staining was performed using 25 µg/mL Hoechst®33342 (Hoechst) (Sigma-Aldrich, St. Louis, MO, USA). Fluorescent images were captured using a fluorescence inverted microscope (DMi8) (Leica Camera AG, Wetzlar, Germany). Exposure time and gain were fixed, and an image of each embryo was photographed with the nucleus in focus. Fluorescence intensity was quantified using ImageJ software1.53f51 (National Institutes of Health; http://imagej.nih.gov/ij/). The ratio of the amount of PDH fluorescence to the amount of pPDH fluorescence was then calculated to obtain the PDH activity (PDH/pPDH). Similarly, ROS level per embryo was evaluated by quantification of fluorescence intensity.

### Cell counting based on immunostaining

After removing the zona pellucida in acidic Tyrode’s solution (pH 2.5), the blastocyst-stage embryos were fixed with phosphate-buffered saline (PBS) containing 4% (w/v) paraformaldehyde (FUJIFILM Wako Pure Chemical Corporation) and permeabilized in PBS containing 0.02% (v/v) polyvinyl alcohol (FUJIFILM Wako Pure Chemical Corporation) and 0.2% (v/v) Triton X-100 (FUJIFILM Wako Pure Chemical Corporation) for 1 h at 20 °C. Subsequently, the embryos were blocked for 1 h with PBS containing 1% (w/v) fetal bovine serum (R&D Systems, Minneapolis, MN, USA) and 0.1% (v/v) Triton X-100. The following primary antibodies were used for analysis: anti-CDX2 (ab76541, rabbit monoclonal, 1:400; Abcam, Cambridge, UK) and anti-Oct3/4 (sc-5279, mouse monoclonal, 1:100; Santa Cruz Biotechnology, Dallas, TX, USA). The temperature for the primary antibody reaction varied depending on the target protein: overnight at 37 °C for CDX2 and overnight at 4 °C for Oct3/4. Alexa Fluor 488 goat anti-mouse IgG (A11001, polyclonal, 1:400; Invitrogen) and Alexa Fluor 555 goat anti-rabbit IgG (A21428, polyclonal, 1:400; Invitrogen) were used as secondary antibodies. All antibodies were diluted in the blocking solution. The embryos were incubated with the secondary antibodies for 30 min at 20 °C in the dark. DNA was counterstained with 25 µg/mL Hoechst 33342 (Sigma-Aldrich) for 5 min at 20 °C. Embryos were mounted on glass slides using the VECTASHIELD Mounting Medium (Vector Laboratories, Burlingame, CA, USA). Fluorescence signals were visualized using a DMi8 fluorescence microscope and the LAS X software (Leica Camera AG). The ratio of marker protein-positive blastomeres to all blastomeres (Fig. 2A and B; blue: DNA) within an embryo was analyzed by manually counting the blastomeres in the immunofluorescence images.

### TdT-mediated dUTP nick end labeling (TUNEL) assay

DNA fragmentation in apoptotic cells was detected by TUNEL assay using an In Situ Cell Death Detection Kit, Fluorescein (Roche Diagnostics, Basel, Switzerland). Blastocyst-stage embryos were fixed in 4% paraformaldehyde in PBS for 1 h and permeabilized in PBS/polyvinyl alcohol containing 0.1% (v/v) Triton X-100 and 0.1% (w/v) sodium citrate for 5 min on ice. After washing with PBS/polyvinyl alcohol, the embryos were incubated in microdroplets of the TUNEL reaction mixture containing terminal deoxynucleotide transferase enzyme and fluorescein dUTP for 1 h in a humidified 5% CO_2_ atmosphere at 37 °C in the dark. Positive controls were treated with DNase I incubation mix (200 U/mL; Promega) for 15 min to detect strand breaks using the TUNEL assay. The embryos were mounted using VECTASHIELD Mounting Medium with DAPI (Vector Laboratories), and fluorescence signals were visualized as described above. Each embryo was analyzed to determine the proportion of TUNEL-positive blastomeres (green nuclei) to all blastomeres within the embryo.

### Trophoblast stem (TS) cell culture

TS cells were isolated from blastocysts and cultured as previously described^7,24,25^. Briefly, feeder cells, particularly mouse embryonic fibroblasts, were mitotically inactivated in a cell culture plate using mitomycin C (Sigma-Aldrich). On the following day, blastocysts derived from IVF embryos or mtB-M embryos were placed onto cell culture plates coated with feeder cells in TE medium: RPMI 1640 (Thermo Fisher Scientific) supplemented with 20% fetal bovine serum (FBS), 1 mM sodium pyruvate, 100 µM β-mercaptoethanol, 2 mM L-glutamine, 100 U/mL penicillin, and 100 µg/mL streptomycin)^25^. When the outgrowth reached a suitable size, it detached and dissociated thoroughly. After 4–5 days, colonies of outgrown cells with diameters of 200 µm were separated by treatment with 0.1% trypsin (Nacalai Tesque) and passaged. Apoptosis-positive colonies were detected using CellEvent™ Caspase-3/7 (Thermo Fisher Scientific) according to the manufacturer’s instructions. Briefly, after washing with PBS (-), the cell colonies were treated with CellEvent™ Caspase-3/7 diluted with 5% FBS-PBS (1:250) and incubated for 30 min at 20 °C in the dark. DNA was counterstained with 25 µg/mL Hoechst 33342 for 10 min at 20 °C. Cycloheximide diluted in TE medium (1:200) was used as a positive control.

### Aggregation of mtB-M and tetraploid embryos

To produce tetraploid mouse embryos, diploid embryos were electroshocked as previously described^9^. Two-cell-stage embryos at 24 h after insemination were placed between two gold electrode fusion chambers filled with M2 medium and electroshocked twice at 150 V with a pulse duration of 50 μs. After electrostimulation, the embryos were washed in M2 medium and incubated in M16 for 1 h in a humidified atmosphere of 5% CO_2_ at 37 °C. The presence of tetraploid embryos confirmed successful blastomere fusion. These embryos were cultured until the next cleavage for aggregation with mtB-M embryos. After removing the zona pellucida with acidic Tyrode’s solution, one mtB-M embryo at the four-cell stage and one tetraploid embryo with two blastomeres were gently pipetted with a glass pipette into M2 medium containing 1 mg/mL phytohemagglutinin P (Sigma-Aldrich) at 20 °C (Fig. 4, A and B, and Movie S2). Strongly aggregated mtB-M embryo/tetraploid embryos were washed in M16 medium and incubated in a humidified atmosphere of 5% CO_2_ at 37 °C.

### Production of the fetus from an aggregated embryo

The aggregated embryos that developed to the blastocyst stage 96 h after insemination were transferred to the uterine horns of female recipient mice at day 2.5 of pseudopregnancy. To maintain pregnancy, 1.5 mg of progesterone (ASKA Pharmaceutical Co., Ltd.) was subcutaneously administered to the back near the uterus of the transplanted recipient mouse two days before the birth date. At E19.5, 14 fetuses were surgically removed from the uterus, and neonatal resuscitation was attempted by stimulating the limbs or tail. We confirmed whether spontaneous breathing occurred and subsequently euthanized and removed target organs (brain, heart, spleen, kidney, testis, ovary, tongue, lung, liver, pancreas, fetal membrane, umbilical cord, and placenta), placing them in PBS. The samples were stored at  − 20 °C in PBS for DNA isolation or in RNAlater (Sigma-Aldrich) for RNA isolation.

### Genomic DNA and total RNA isolation

To extract total DNA containing mtDNA, organs from each fetus were lysed in 500 µL of tissue lysis buffer (150 mM NaCl, 10 mM Tris-HCl [pH 7.5], 2 mM EDTA, and 200 µg/ml proteinase K) containing 0.5% (v/v) sodium dodecyl sulfate and incubated overnight at 37 °C. The lysates were purified using phenol/chloroform extraction and ethanol precipitation. The resulting genomic DNA was then resuspended in distilled water. Based on a previous report^26^ of DNA extraction from a single embryo, one embryo was lysed in 10 µL embryo lysis buffer (50 mM Tris-HCl [pH 8.5], 1 mM EDTA, 0.5% Tween-20, and 200 µg/mL proteinase K). After incubation for 2 h at 55 °C, proteinase K was inactivated for 10 min at 95 °C. Total RNA was isolated from the embryos using the ReliaPrep RNA Cell Miniprep System (Promega) by collecting a cohort of embryos per sample. All DNA and RNA extractions were performed using a kit, according to the manufacturer’s instructions.

### Detection of bovine mtDNA

Primers that specifically amplify mouse or bovine mitochondrial *ND5* were used to detect bovine mtDNA in the tissues of Xenon pups. Genomic DNA was amplified using the Ex Taq Hot Start (Takara Bio, Shiga, Japan) with 0.5 µM of each primer. The PCR profile consisted of an initial denaturing step for 5 min at 95 °C, followed by 35 cycles of 94 °C for 30 s, 62 °C for 1 min, and 72 °C for 1 min, with a final extension step at 72 °C for 2.5 min. For nested PCR, the amplicon was diluted with water (1:20) and the above program was performed for an additional 20 cycles with each specific primer. For tissue samples, the amplified products were mixed with 6 × Gel Loading Dye Purple (New England Biolabs, MA, USA), separated by electrophoresis, and cloned into a pGEM-T Easy Vector (Promega), followed by sequencing on an ABI PRISM 310 Genetic Analyzer (Applied Biosystems, CA, USA). At least three clones per fetus were obtained from three independent fetuses. The sequences were aligned to mouse *Nd5* (NC_005089) and bovine *ND5* (NC_006853.1) sequences.

### Quantitative PCR

After preparing reaction mixtures containing THUNDERBIRD SYBR qPCR Mix (TOYOBO, Osaka, Japan) and 0.5 µM of each primer, qPCR was performed in duplicate using a LightCycler 96 (Roche Diagnostics). To analyze target gene expression, cDNA was synthesized using ReverTra Ace qPCR RT Master Mix (TOYOBO). The thermal cycling conditions consisted of one cycle at 95 °C for 30 s, followed by 55 cycles at 95 °C for 10 s, an annealing temperature corresponding to each primer set for 15 s, and 72 °C for 30 s. The transcript levels were calculated by the ΔΔCt method, with *Gapdh* or *H2afz* as the reference genes for each sample. All primer and probe sequences are listed in Table S4.

### Histological analysis

Lungs from E19.5 pups were fixed with 4% paraformaldehyde and processed for paraffin sectioning. Sections (4 μm thick) were stained with hematoxylin and eosin. Each value of lung (alveolar lumen area, number of alveoli, and area of Clara cell in random 10^5^ µm^2^ regions) were measured using ImageJ software.

### RNA sequencing

The RNA integrity number of total RNA isolated from the lung tissue (control ICR males and females, and Xenon males and females; n = 3 for all groups) was determined using an Agilent 2100 Bioanalyzer (Agilent Technologies, CA, USA) with an Agilent RNA 6000 Nano Kit (Agilent Technologies). RNA-seq libraries were constructed according to the instructions provided in the TruSeq RNA Library Prep Kit (Illumina, San Diego, CA, USA) and sequenced using a HiSeq 4000 platform (Illumina) with 100 bp paired-end sequencing. After checking the read quality using FastQC (Ver. 0.11.7), three bases at the beginning and end of the reads were trimmed using Trimmomatic (Ver. 0.38) if their Phred quality scores were  < 20. Refined reads that were not mapped to the mouse reference genome (RefSeq assembly accession: GCF_000001635.20) were mapped to the bovine mitochondrial genome (RefSeq ID: NC_006853.1) and mouse mitochondrial genome (RefSeq ID: NC_005089.1) using HISAT2 (Ver. 2.1.0). Reads assigned to gene transcripts were counted using featureCounts (ver. 1.6.3). DESeq2 (Ver. 3.4.1) was used to detect DEGs, identified using P-values adjusted by the Benjamini–Hochberg method (*Padj*) < 0.01. Gene expression levels were represented as FPKM, calculated using StringTie (Ver. 1.3.4d). Transcripts with FPKM> 1.0 were used for subsequent gene expression analysis.

### Functional analysis of expressed genes

GSEA was performed using the Broad Institute website (http://www.broadinstitute.org/gsea/index.jsp)^27^ and was conducted on all expressed genes (12,532 genes, FPKM> 1.0). Enriched gene sets with a false discovery rate (FDR) < 0.25 were considered statistically significant. Statistical significance was set at *p* < 0.05.

### Statistical analysis

Statistical comparisons were performed using the Student's* t*-test. For comparisons between three groups, significant differences were detected using the Tukey and Kramer tests. All statistical analyses were conducted in R. Data are expressed as the mean ± standard error of the mean. Statistical significance was set at *p* < 0.05, *p* < 0.01, and *p* < 0.001.

### Supplementary Information

Supplementary Information 1.

Supplementary Information 2.

Supplementary Video 1.

Supplementary Video 2.

## Data Availability

The RNA-seq data generated in this study have been submitted to the DDBJ Data Bank (https://www.ddbj.nig.ac.jp) under accession number DRA012493.

## Abbreviations

DEGs: Differentially expressed genes
FDR: False discovery rate
FPKM: Fragments per kilobase of exon per million mapped reads
GO: Gene ontology
GSEA: Gene set enrichment analysis
ICM: Inner cell mass
IVF: In vitro fertilization
MRC: Mitochondrial respiratory chain
mtB-M embryo: Mice embryo harboring bovine mitochondria
mtDNA: Mitochondrial DNA
non-mtB-M embryo: Mice embryo containing bovine cytoplasm distinct from the mitochondria enrichment region from bovine embryo
mtM-M embryo: Mice embryo with mitochondria derived from another mouse embryo
FBS: Fetal bovine serum
PBS: Phosphate-buffered saline
PCR: Polymerase chain reaction
PDH: Pyruvate dehydrogenase
qPCR: Quantitative real-time PCR
RNA-seq: RNA sequencing
ROS: Reactive oxygen species
SCNT: Somatic cell nuclear transfer
SEM: Standard error of the mean
TE: Trophectoderm
TS cell: Trophoblast stem cell
TUNEL: Transferase-mediated dUTP nick end-labeling
WT: Wild-type
