# Supplementary material for: Dual barrier system against xenomitochondrial contamination in mouse embryos
Source: Sci Rep. 2023 Dec 27;13:23058. doi: 10.1038/s41598-023-50444-2 (PMC10754889; doi:10.1038/s41598-023-50444-2)
Supplement: Supplementary file 1 — Supplementary Information 1. [file 41598_2023_50444_MOESM1_ESM.docx]

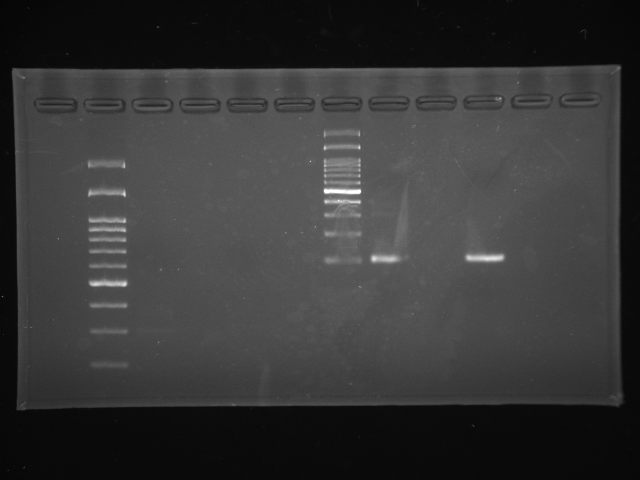

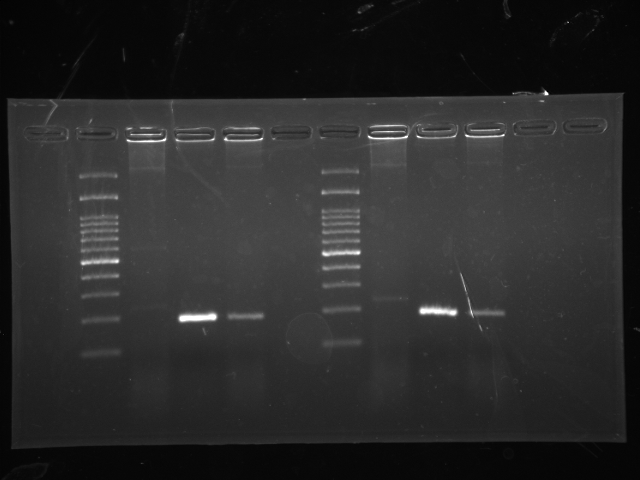


**Marker**

**Marker**

**Xenon**

**Bovine**

**genome**

**Mouse**

**genome**

**bovine**

***ND5***

**(194 bp)**

**Fig4E**

**Xenon**

**Bovine**

**genome**

**Mouse**

**genome**

**mouse**

***Nd5***

**(108 bp)**

**Fig4E**


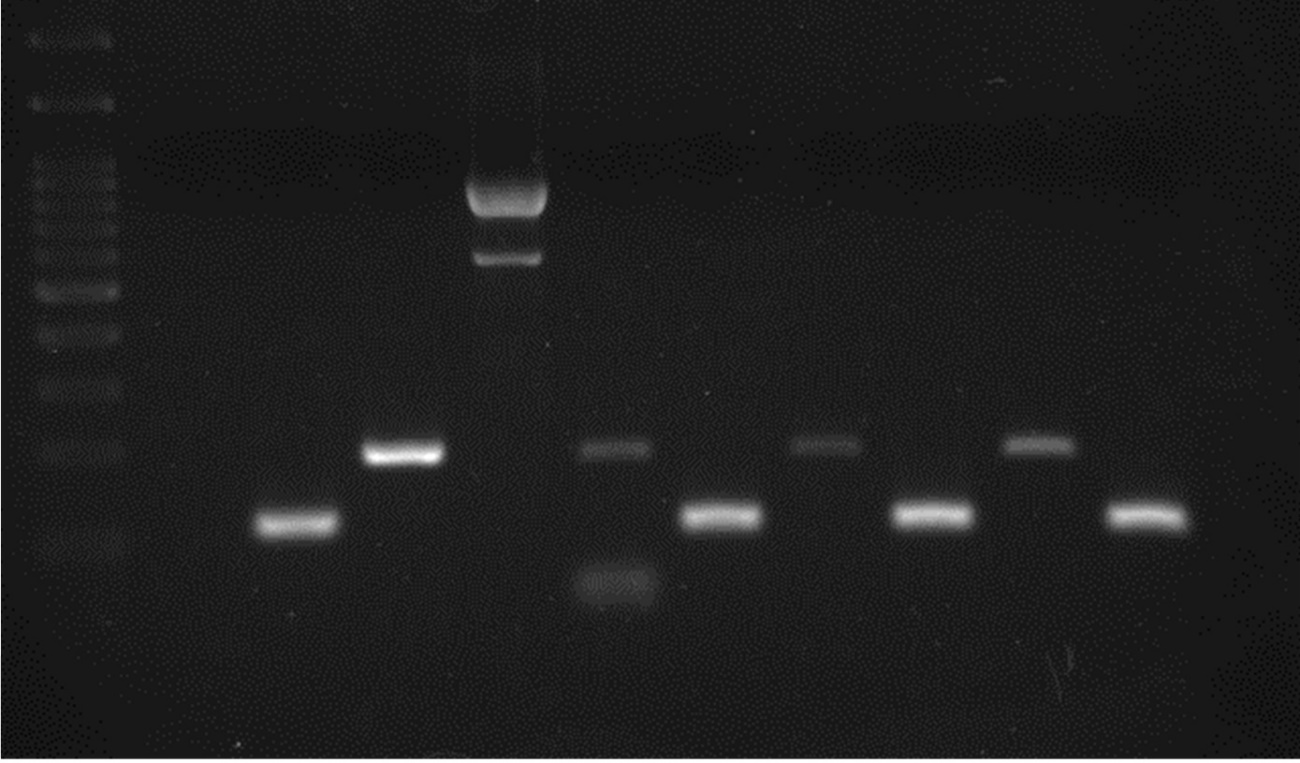


**Primer B M B M B M**

**mtB-M-derived**

**cells**

**Mouse**

**genome**

**Bovine**

**genome**

**Fig3B**

**Bovine *ND5***

**(194 bp)**

**mouse Nd5**

**(108 bp)**





**Fig3B**

**Marker**

**Samplewells**


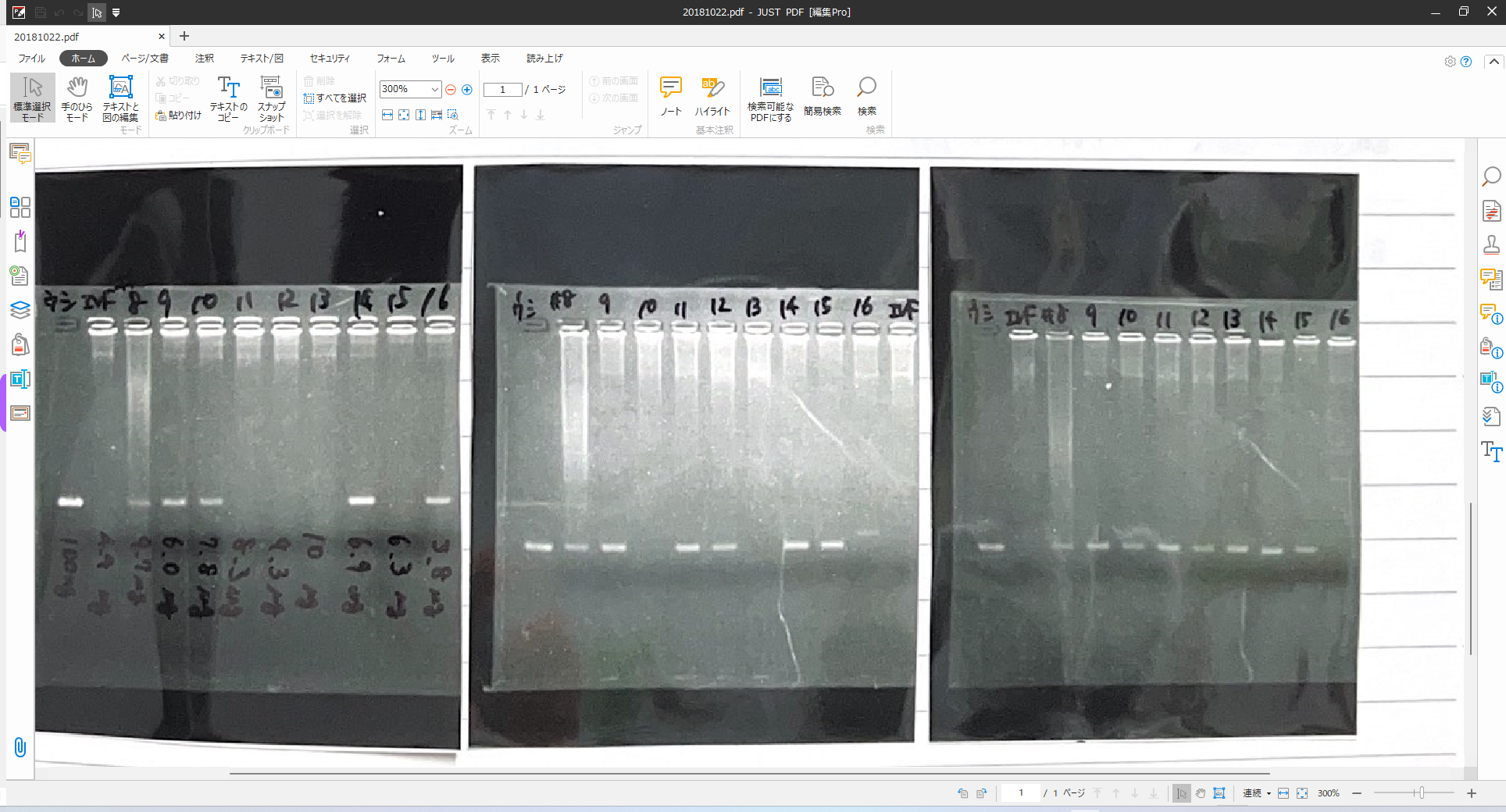


**Figure S5**

**Bovine *ND5***

**(194 bp)**

**Bovine**

**genome**

**Mouse**

**genome**

**XM1**

**XF1**

**XM2**

**XF2**

**XF3**

**XM3**

**#1**

**#2**

**#5**

**#6**

**#3**

**#4**

**#7**

**in Fig. 5A**

**in Fig. 5B**

―

―

―

―

―
