# Supplementary material for: Dual barrier system against xenomitochondrial contamination in mouse embryos
Source: Sci Rep. 2023 Dec 27;13:23058. doi: 10.1038/s41598-023-50444-2 (PMC10754889; doi:10.1038/s41598-023-50444-2)
Supplement: Supplementary file 2 — Supplementary Information 2. [file 41598_2023_50444_MOESM2_ESM.docx]

**Supplementary Information**

Dual barrier system against xenomitochondrial contamination in mouse embryos

Masaya Komatsu, Hikaru Takuma, Shun Imai, Maiko Yamane, Masashi Takahashi, Takuto Ikegawa, Hanako Bai, Hidehiko Ogawa and Manabu Kawahara

**This PDF file includes:**

**Supplemental figures S1-S5**

**Supplemental tables S1-S4**

**Separate files:**

**S1 Movie. Procedure of manipulation for mtB-M embryo.**

**S2 Movie. Aggregation of mtB-M and tetraploid embryos.**

**Figure S1.
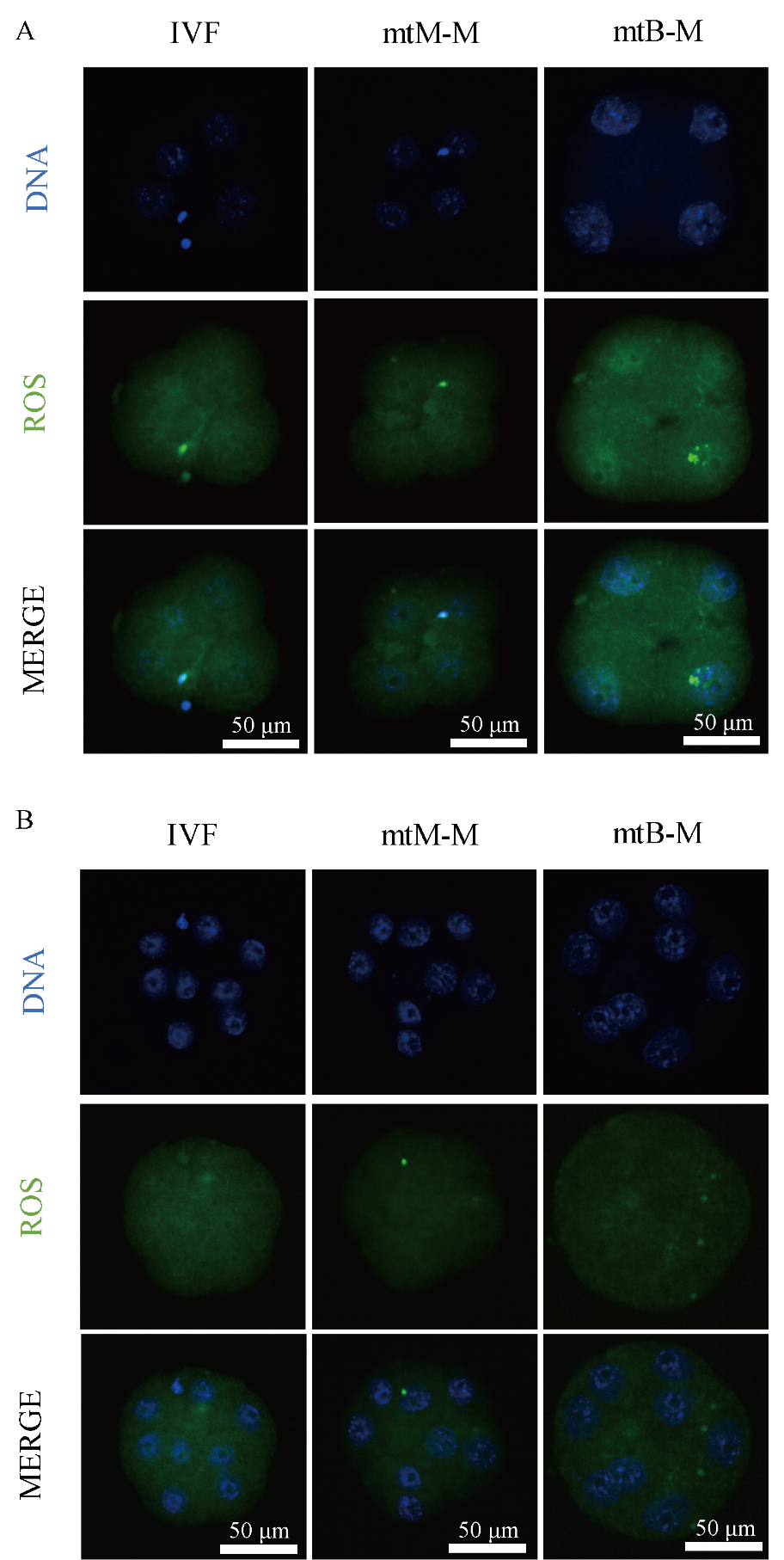
 Representative images for Reactive Oxygen Species (ROS) production in embryos. (A)** 4-cell stage. **(B)** 8-cell stage. mtM-M: mouse embryos with mitochondria from another mouse embryo. mtB-M: mouse embryos introduced with bovine mitochondria.

**Figure S2. Histogram of embryonic ATP content at each cell stage.** Each histogram represents the distribution of ATP content per embryo (pmol) from the 1-cell (1C) stage to the blastocyst stage. IVF embryos (blue); 1C: n = 50, 2C: n = 52, 4C: n = 51, 8C: n = 53, morula: n = 51, and blastocyst: n = 51). mtB-M embryos (red); 1C: n = 50, 2C: n = 53, 4C: n = 51, 8C: n = 53, morula: n = 51, and blastocyst: n = 51). Among these, values within mean ± 1 standard deviation were selected and analyzed for each cell stage in Fig. 1E.

**Figure S**
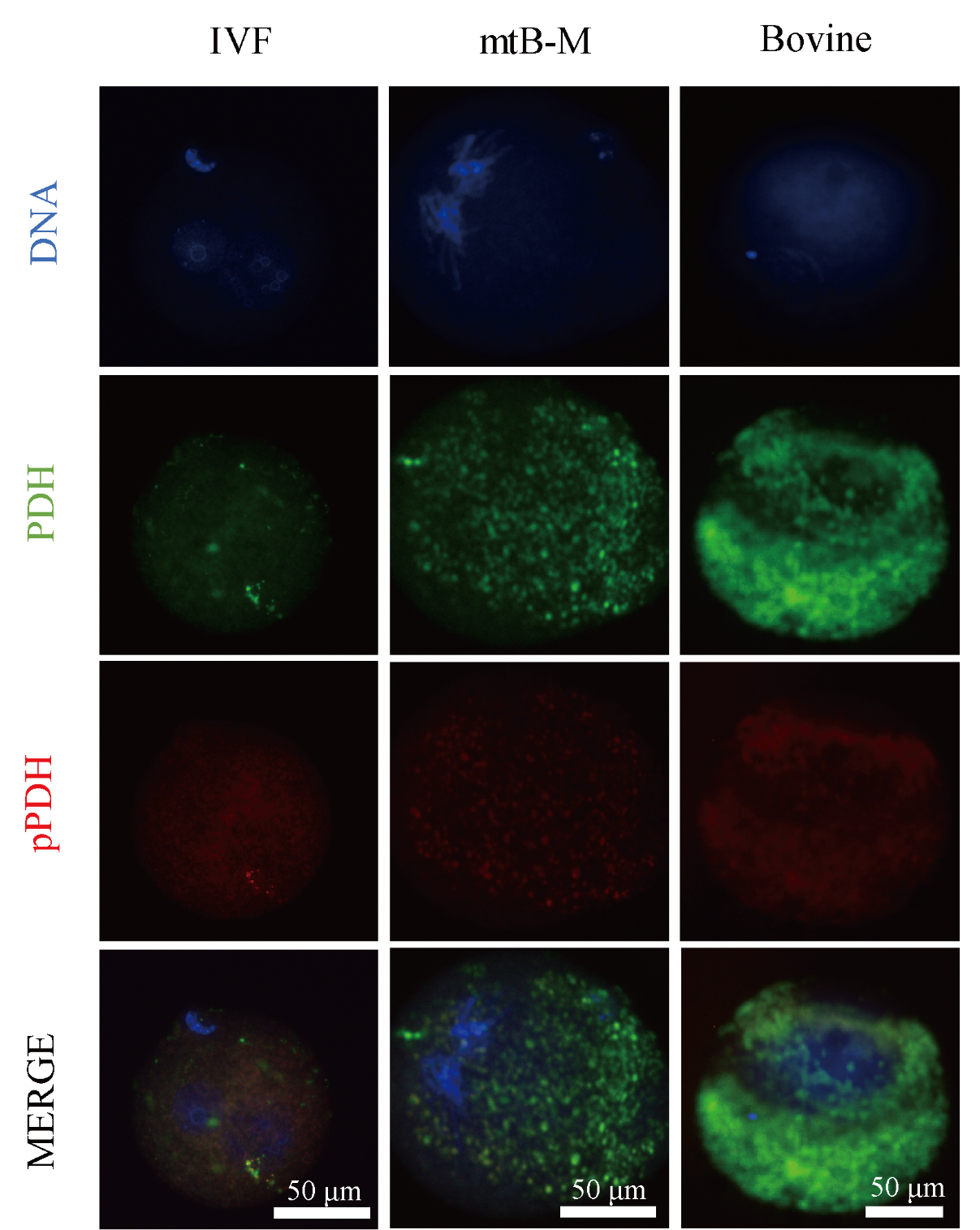
**3. Representative images for PDH and pPDH immunostaining in 1-cell stage embryos.** mtB-M: mouse embryos introduced with bovine mitochondria. Bovine: bovine embryos at the 1-cell stage.

**Figure S4. Histological analysis of Xenon lungs at E19.5. (A)** Standardised weights of primal organs divided by body weight (IVF control, n = 5; Xenon, n = 3). **(B)** Representative sections (haematoxylin/eosin) of the left lung lobes in IVF controls and Xenon pups. **(C)** The number of alveoli and the alveolar lumen and Clara cell area per 10^5^ µm^2^ of each lung section (IVF control, n = 5; Xenon, n = 3)

**
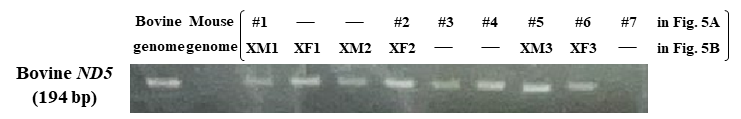
**

**Figure S5. Detection of bovine *ND5* in Xenon lungs by PCR with specific primer.** The lungs of fetus ID#1, 2, 5, and 6 in Fig. 5A correspond to XM1, XF2, XM3, and XF3 in Fig. 5B. Bovine *ND5* in both XF1 andXM2 lungs were not quantified in Fig. 5A.

**Table S1.** **Developmental competence of mtB-M embryos to term.**

^a-b^: Values with different letters indicate statistical significance (*p* < 0.05).

※1: "mtM-M" embryos represent mouse embryos with mitochondria from another mouse embryo.

※2: "non mtB-M" embryos represent the embryos fused with cytoplast distinct from the mitochondria enrichment region from bovine embryos.

**Table S2.** **Viability of mtB-M embryos aggregated with tetraploid embryos.**

^a-b^: indicates statistical significance (*p* < 0.05).

**Table S3. Read number mapped to the mouse and bovine mtDNA genomes.**

CM, control male; CF, control female; XM, Xenon male; XF, Xenon female.

**Table S4. The primer and probe sets used for the PCR.**
